# Creole Prosodic Systems Are Areal, Not Simple

**DOI:** 10.3389/fpsyg.2021.690593

**Published:** 2021-10-27

**Authors:** Kofi Yakpo

**Affiliations:** Department of Linguistics, The University of Hong Kong, Hong Kong, Hong Kong SAR, China

**Keywords:** simplification, prosodic system, stress, tone, creole, linguistic ecology, language contact, areal convergence

## Abstract

This study refutes the common idea that tone gets simplified or eliminated in creoles and contact languages. Speakers of African tone languages imposed tone systems on all Afro-European creoles spoken in the tone-dominant linguistic ecologies of Africa and the colonial Americas. African speakers of tone languages also imposed tone systems on the colonial varieties of English, French, Spanish, and Portuguese spoken in tonal Africa. A crucial mechanism involved in the emergence of the tone systems of creoles and colonial varieties is stress-to-tone mapping. A typological comparison with African non-creole languages shows that creole tone systems are no simpler than African non-creole tone systems. Demographic, linguistic, and social changes in an ecology can lead to switches from tone to stress systems and vice versa. As a result, there is an areal continuum of tone systems roughly coterminous with the presence of tone in the east (Africa) and stress in the west (Americas). Transitional systems combining features of tone and stress converge on the areal buffer zone of the Caribbean. The prosodic systems of creoles and European colonial varieties undergo regular processes of contact, typological change and areal convergence. None of these are specific to creoles. So far, creoles and colonial varieties have not featured in work on the world-wide areal clustering of prosodic systems. This study therefore aims to contribute to a broader perspective on prosodic contact beyond the narrow confines of the creole simplicity debate.

## 1. Introduction

Creolization is said to involve the simplification of input structures (for a thematic overview, see Ansaldo et al., 2007). One such structure is tone, which has been argued to constitute a feature that gets lost or is starkly reduced during language contact and creolization (e.g., Heine, 1978, 220; Salmons, 1992; Sebba, 1997, 49; McWhorter, 1998, 793; Kusters, 2003, 343; Trudgill, 2010, 309; Sessarego, 2020, 3). I propose that creolization has neither led to the elimination nor the simplification of tone systems.

Instead, creoles feature prosodic systems ranging from tone to stress, and to mixed systems incorporating both. The same holds for European colonial varieties (varieties of English, French, Spanish, and Portuguese spoken in Africa and the Americas). These are generally left out of discussions about creole prosody but are essential for developing a general typology of prosodic contact outcomes.

The prosodic systems of creoles and colonial varieties (contact prosodic systems) have developed tone or stress systems in accordance with the linguistic factor of areal typology (dominance of tone vs. stress in the ecology), the cognitive factor of psycholinguistic dominance (recipient vs. source-language agentivity), and social factors in their specific linguistic ecologies (the demographic proportion and social stratification of speakers of tone and stress-only languages) (Bordal Steien and Yakpo, 2020; Yakpo, 2020).

Further, there is an east-west, tone-stress continuum from Africa to the Americas. Contact prosodic systems in African ecologies and in isolated ecologies of the Americas (e.g., in the Amazonian region of Suriname) feature tone systems. In this, they reflect the prosodic proclivities of their adstrates (African languages presently spoken by the multilingual speakers of the creoles) and substrates (African languages once spoken alongside the creoles). The prosodic systems of most creoles and colonial varieties spoken in the Americas have, in turn, converged toward the stress-only systems of their European lexifiers (lexicon-providing languages) and superstrates (socially dominant languages, whether lexifier or not) but still maintain marginal tonal features (for detailed creolist definitions of adstrate, substrate, lexifier, and superstrate, see Yakpo, 2017a, b, 53, 227–229).

Afro-European creoles and colonial varieties therefore constitute no exception to the world-wide tendency of prosodic systems to cluster areally (Matisoff, 2001; Gussenhoven, 2004, 42–45; Clements and Rialland, 2007, 74). So far, creoles and colonial varieties have not featured in studies on the areal clustering of prosodic systems (e.g., Maddieson, 2013). This study therefore aims to contribute to a broader perspective on prosodic contact beyond the narrow confines of creole linguistics. The results of this study also complement and support the stratal-areal contact model proposed in earlier work (Yakpo, 2017a), which explains long-term contact outcomes in creoles spoken in the multilingual linguistic ecologies of Africa and the Americas.

Arguments that creole grammars and prosodic systems are simpler than those of non-creoles are based on the concept of ‘bit complexity’ (DeGraff, 2001, 284–285), which comes down to a simplistic measure of ‘more overt material = more complex’. Even from the perspective of bit complexity, creole tone systems are *more complex* than those of the colonial varieties of English, French, Spanish, and Portuguese (section 3.1 and section 4). This is due to social factors that impede the same amount of innovation and areal diffusion of tonal features to the colonial varieties as to the creoles (section 6).

I identify three cognitive-typological mechanisms that drive the creation of contact prosodic systems in the encounter of tonal substrates and adstrates, and lexifiers and superstrates that make use of stress. These are stress-to-tone mapping, paradigmatization, and idiosyncratization. Neither the mechanisms themselves, nor their outcomes involve simplification. Instead, contact prosodic systems acquire their properties from ‘typological matching’ (Mufwene, 1996, 2001; Aboh and Ansaldo, 2007) between the features of the input languages in a specific linguistic ecology. Crucially, the acoustic and phonological realization of tone in the adstrates and substrates is matched with, and where compatible, grafted on the corresponding realization of stress in the lexifier.

A few additional definitions are in order before proceeding. In languages with stress, words or phrases are associated with metrical structure that is determined with respect to the position of a stressed syllable meeting two criteria. The first is obligatoriness: Every word or phrase has *at least* one syllable marked for primary stress, the highest degree of metrical prominence. The second criterion is culminativity: Every word or phrase has *no more than* one syllable marked for the highest degree of metrical prominence (Hyman, 2006, 231). The acoustic correlates of stress are language-specific but usually involve a combination of the cues of length, loudness, vowel quality, and pitch variations over the stress-bearing syllable. The pitch contours of utterances in languages that are non-tonal are composed of intonational pitch accents anchored to stressed syllables, and boundary tones associated with the edges of phrases and utterances. I henceforth refer to languages with stress, but no tone (some tone languages also have stress) to ‘stress-only’ languages. All the lexifiers and superstrates of the Afro-European creoles and colonial varieties (English, French, Spanish, Portuguese, and Dutch) are exclusively stress-only languages.

In languages with tone, pitch features are instead part of (at least some) morphemes and therefore part of the lexical realization of morphemes together with vowels and consonants (Welmers, 1959, 2; Hyman, 2001, 1367). The lexical tones of morphemes can therefore only be changed by specific rules akin to those which alter the segmental realization of morphemes. All the substrates and adstrates of the Afro-European creoles and colonial varieties treated here are tone languages.

Pitch is nevertheless employed in both stress-only and tone systems for the non-lexical purposes of intonation, that is, for marking phrase boundaries and expressing pragmatic functions like emphasis, focus, and asking questions (e.g., Downing and Rialland, 2016). However, stress-only systems use pitch distinctively *only* in these phrasal ways.

A concept that is equally important for the ensuing discussion is Van Coetsem’s psycholinguistic metaphor of ‘agentivity’ (1988, 2000). The language with agentivity provides features to the contact language or variety. In the case of recipient language agentivity, features from a non-native source language are transferred to the speaker’s dominant or native recipient language by *borrowing*. Source language agentivity represents the opposite case. The speaker uses a non-native recipient language, and features from her dominant or native source language are transferred to the recipient language by *imposition* (termed ‘interference’ in earlier work, see Weinreich, 1953; Thomason and Kaufman, 1988). Source language agentivity therefore manifests itself as substratal (in cases of shift from the source language to the recipient language) or adstratal (in cases of maintenance of the source and recipient languages) areal influence on the recipient language.

The Afro-European contact scenarios treated here all constitute cases of source language agentivity. The importance of this cannot be stressed enough. All sources that claim tone loss and reduction in language contact and creolization fail to make the distinction between the two transfer types of recipient language and source language agentivity. They therefore fail to identify the directionality of change in contact prosodic systems, thereby jumping to the logically flawed conclusion that stress trumps tone (section 5).

The paper is organized as follows. I first present an analysis of the contact prosodic systems of Pichi and Guyanese Creole which occupy different spaces on the continuum of contact outcomes between African tone systems and European stress-only systems (section 2). I then provide evidence for an areal distribution of the prosodic systems of Afro-European creoles and colonial varieties along an east to west, Africa to the Americas axis (section 3). This distribution roughly corresponds to the presence of tone in Africa and stress in the Americas, with transitional prosodic systems in the Caribbean, the areal buffer zone between the two. I then identify three concrete mechanisms with potential for generalization, which were involved in the emergence of contact prosodic systems with tone (section 4). Finally, I compare features of tonal Afro-European creoles, which have been claimed to constitute simplification, with those of tonal non-creole languages in Africa (section 5). I show that there is no evidence for simplification in the tone systems of Afro-European creoles. The study is concluded with some general remarks on the role of social factors in the differential outcomes of prosodic contact (section 6).

## 2. Afro-European Creoles Can Have Tone, Stress, and a Mix of Both

Two case studies follow of the prosodic systems of the English-lexifier creoles Pichi (Equatorial Guinea) (section 2.1) and Guyanese Creole (Guyana) (section 2.2). The two languages belong to the linguistic family of Afro-Caribbean English-lexifier Creoles with shared ancestry in a (number of) 17th century protolanguage(s) in the Caribbean and West Africa (Hancock, 1986, 1987; Smith, 1987). Pichi and Guyanese Creole occupy different sections of an areal continuum of contact prosodic systems across the Afro-Atlantic (see section 3). Pichi has a tone system and Guyanese Creole has a mixed system featuring tone and stress.

### 2.1. A Creole With Tone: Pichi

The English-lexifier creole Pichi is spoken on the island of Bioko, Equatorial Guinea. A detailed description of the tone system of Pichi including acoustic evidence is provided by Yakpo (2019a, 37–60). The following sections summarize relevant aspects of the system.

#### 2.1.1. Tones, Tone Patterns, and Minimal Pairs

Pichi has an ‘equipollent’ (Hyman, 2011a) 2-tone system with a High (H) and a Low (L) tone. This means that /H/ and /L/ are both lexically specified and phonologically activated, and subjected to tonal rules and processes (see section 2.1.2). There is no acoustic evidence for stress.

The Pichi prosodic lexicon is etymologically layered due to the mechanism of stress-to-tone mapping that converted English stress to tone (section 4.1). Most English-sourced words, which constitute the majority of the lexicon, feature an obligatory (at least one) and cumulative (at most one) H tone. The most frequent patterns are an /H/ over a monosyllabic word (54% of my corpus) e.g., *áks* ‘ask’, an /H-L/ sequence over a disyllabic word (23%), e.g., *húmàn* ‘woman’, and an /L-H/ sequence over a disyllabic word (14%), e.g., *grèví* ‘gravy’. More diverse patterns without the restriction of obligatory and cumulative /H/ are present in a few English-sourced words, e.g., *ápás* /H-H/ ‘after’ (<Eng. *half past*), and African-sourced words, e.g., *nyɔ́ní* /H-H/ ‘ant’ (<Mende *yɔ́ní*), *òkóbó* /L-H-H/ ‘impotent man’ (<Yoruba *òkóbó*). A further pattern consists of an /L/ tone in English-sourced monosyllables whose etymons normally remain unstressed, e.g., *bìn* /L/ ‘past tense marker’ (<*been*), *dì* /L/ ‘definite article’ (<*the*), or African-sourced monosyllables with the same specification for /L/ tone, e.g., *nà* /L/ ‘general locative preposition’ (<Igbo *nà* ‘general locative preposition’; also found as a reflex of Proto-Niger-Congo ^∗^*na* in hundreds of African substrate and adstrate languages).

Ideophones and interjections of African or unknown origin feature more diverse word-tone patterns, often due to lexicalized duplication and triplication, e.g., *ékìé* /H-L-H/ ‘expression of counter-expectation’, *kɔ́ngkɔ́ngkɔ́ng* /H-H-H/ ‘requesting entry’, *ményéményé* /H-H-H-H/ ‘whine in a childlike fashion’, *gbògbògbò* /L-L-L/ ‘hastily’, *kàmúkàmú* /L-H-L-H/ ‘sight of buttocks moving’, and *súkútúpàmpà* /H-H-H-L-L/ ‘in a cheap and mean fashion’.

Some monosyllabic roots are distinguished from each other by tone alone, see Table 1. In conformity with a general pattern, function words tend to be L-toned, while the corresponding content words are mostly H-toned.

**TABLE 1:** Monosyllabic tonal minimal pairs in Pichi.

H tone	Gloss	L tone	Gloss
*báy*	‘buy’	*bày*	‘by’
*bɔ́t*	‘hit with the head’	*bɔ̀t*	‘but’
*dé*	‘day; there’	*dè*	‘IPFV’
*dí*	‘this’	*dì*	‘DEF’
*gó*	‘go’	*gò*	‘FUT; POT’
*lɛ́k*	‘(to) like’	*lɛ̀k*	‘like’
*só*	‘like this; sew; show’	*sò*	‘so’
*wét*	‘wait’	*wèt*	‘with’

Pichi also has a few disyllabic minimal pairs, see (1) in Table 2. We also find the maximal number of possible tone patterns over disyllabic words, see (2). A phrasal tonal minimal pair is given in (3), where *òpìn-yày* ‘open-eye(s) = cultivated’ has undergone the tonal derivation of compounding (see section 2.1.2). Abbreviations and glossing conventions are listed and explained at the end of this article.

**TABLE 2:** Multisyllabic tonal minimal pairs in Pichi.

	Item	Gloss	Item	Gloss
(1)	*kàtá /*L-H/	‘catarrh’	*kátà /*H-L/	‘scatter’
	*pàpá /*L-H/	‘father’	*pápà /*H-L/	‘potato’
(2)	*fíbà* /H-L/	‘fever’	*nyɔ́ní* /H-H/	‘ant’
	*wàtá* /L-H/	‘water’	*bàtà* /L-L/	‘buttocks’
(3)	*ópìn yáy /*H-L H/	‘open (an) eye’	*òpìn-yáy /*L-L-H/	‘cultivated’

#### 2.1.2. Tonal Processes and Grammatical Tone

Pichi tonal processes are operative within prosodic domains of various sizes (see Yakpo, 2019a, 46–57 for details and pitch traces of examples provided in this section). Processes include tonal plateauing when the L-toned syllable of a disyllabic verb with an H-L pattern is hemmed in by the left-adjacent H and a right-adjacent H of a following object, as in *prɔ́mìs mí* /H-L H/ → *prɔ́****mís*** *mí* [H-**H** H] ‘promise me’. Pichi also features downdrift (indicated by ↓H), which causes an H to be lowered by a preceding L tone, as in *yɛ́stà****dé*** [H-L-**↓H**] ‘yesterday’. In a series of adjacent H tones, we find downstep (also indicated by ↓H): Each H tone is lowered successively in relation to the preceding one, as in *wákà sén* ***sén sén*** [H ↓**H** ↓**H**] ‘walk same same same = walk exactly in one line’. Pichi also features pitch or register raising for focal emphasis when all H tones of a focused constituent are raised a notch higher (also see 2.2.3, 3.1, and 4.3).

There are also instances of grammatical tone, i.e., processes restricted to the context of a specific morpheme or construction (Rolle, 2018). Tone floating and contour tone formation take place when the H-toned subjunctive marker (a complementizer) *mék* /H/ ‘SBJV’ occurs left-adjacent to the monosyllabic personal pronouns *à* /L/ ‘1SG.SBJ’ and *è* /L/ ‘3SG.SBJ’. The final consonant of *mék* is generally not pronounced and this leads to a vowel hiatus and to further deletion of the vowel of *mék*. In the process, the H tone of *mék* is floated and linked to the L-toned syllable of the personal pronouns *à* and *è*, i.e., *mâ* /HL/ ‘SBJV.1SG.SBJ’ and *mê* /HL/ ‘SBJV.3SG.SBJ’. The resulting portmanteau morphemes and contour tone are so common (the two words/tones are almost always merged), that the contour tone may be seen to be phonologized, i.e. *má* /H/ ‘mother, madam’ vs. *mâ* /HL/ ‘SBJV.1SG.SBJ’.

The inflectional expression of the grammatical relations of subject, object, and possessive case involves the use of tonal suprafixation with personal pronouns (see Yakpo, 2019a, 128), see Table 3.

**TABLE 3:** Suprafixation with personal pronouns in Pichi.

Category expressed	Suprafix
Subject and possessive case	L tone
Object case and emphasis	H tone

An example of case assignment in 1SG pronouns (object vs. possessive case) via tonal ablaut is given in (1). Note that in cases of clash between subject case and emphasis, the latter series of pronouns wins out, e.g., ***mí*** *nó sàbí* ‘I [EMP] don’t know’ vs. *à nó sàbí* ‘I don’t know’.


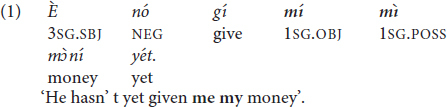


The use of grammatical tone also characterizes tonal derivation incompounding and morphological reduplication. The H tone of the dependent is replaced by an L tone, while the head retains its original tone pattern; compare *wách* /H/ (to) ‘watch’ and *mán* /H/ ‘man’ → ***wàch****-mán* /**L**-H/ ‘watchman’, or ***wàch****-wách* /**L**-H/ ‘to continuously/repeatedly watch’. An example involving a disyllabic dependent is *bɛ́rìn* /H-L/ ‘bury’ and *grɔ́n* /H/ ‘ground’ → ***bɛ̀**rìn-grɔ́n* /**L**-L-H/ ‘burial ground’ (also see Yakpo, 2012).

Thirdly, Pichi features a tone-conditioned suppletive allomorphy, a cross-linguistically rare or at least underreported phenomenon (Paster, 2006). Pichi has two pronominal variants that both instantiate (direct and indirect) object case. The variants are the clitic object pronoun =*àm* ‘3SG.OBJ’ and the phonologically independent and emphatic pronoun *ín* ‘3SG.EMP’. The clitic =*àm* ‘3SG.OBJ’ is the default form used in all licit contexts. Hence =*àm* is the only possible option if the host verb features a word-final consonant (2) or word-final H-toned vowel (3).


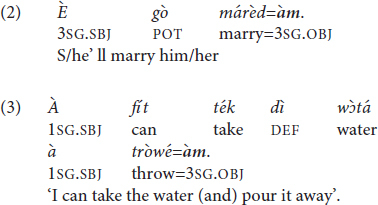


Examples (4) and (5) featuring the independent pronoun *ín* ‘3SG.EMP’ are therefore ungrammatical:


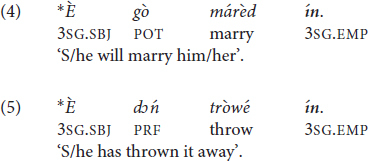


The use of the allomorph =*àm* is, however, also ungrammatical if the host features a word-final L-toned vowel (6). Pichi tonotactics disallow string-adjacent identical tones in the same phonological word, hence in this case ^∗^V̀V̀ >> V̀CV̀, V́V̀ (string-adjacent H tones are also banned but this is not relevant here). The corresponding examples are ^∗^(6) >> (2), (3) (for additional layers of rules, see Yakpo, 2019b, 206–212). The restriction is therefore a manifestation of the Obligatory Contour Principle (OCP) (Leben, 1973). In order to avoid a breach of the OCP, the independent and emphatic pronoun *ín* is recruited when the verb features a word-final L-toned vowel (7).


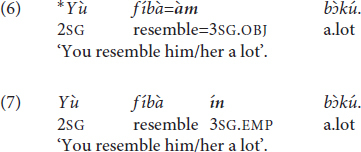


### 2.2. A Creole With Stress and Tone: Guyanese Creole

The following description of the prosodic system of Guyanese Creole examines aspects of the analyses by Devonish (1989, 2002; and pers. comm.) and Devonish and Thompson (2010). My interpretation of the data is that Guyanese Creole has a mixed prosodic system featuring both lexical tone and stress (section 2.2.1 and section 2.2.2). Guyanese Creole additionally features residual tone in ideophones (section 2.2.1 and section 3.2).

#### 2.2.1. Stress, Tone, Prosodic Patterns, and Minimal Pairs

Guyanese Creole makes use of tone and stress. The most reliable indicator of stress in Guyanese Creole is a quantity contrast: Stressed syllables are generally longer than unstressed ones. Stress appears to be assigned lexically, but the nature of stress placement is not elucidated fully in the sources. I therefore focus on the pitch-related aspects of the prosodic system. Devonish and Thompson (2010, 9) refer to Guyanese Creole as a ‘restricted’ tone language. I assume that the language has a privative contrast between /HL/ and Ø (i.e., zero or toneless) in the majority lexicon. Hyman (2011a, 191) employs the term ‘privative’ to characterize a binary contrast (/HL, Ø/ in the case of Guyanese Creole), in which only one tone (i.e., /HL/) is phonologically active, i.e., ‘invoked by the language’s constraints or rules’. The pitch traces contained in Devonish (2002) also indicate interpolation (gradual transitions between pitch peaks). Further, the presence of the HL contour is obligatory and culminative in lexical words, and there are positional restrictions on its occurrence. Tone systems like that of Guyanese Creole are also called ‘sparse’, a term I will use from now on (Gussenhoven, 2004, 34–35; Hyman, 2011b, 235).

The HL lexical tone is assigned independently of stress and may or may not coincide with the stressed syllable. There are various types of output prosodic patterns. One group consists of toneless (Ø) monosyllabic clitics that are realized as [L]. These form tonal minimal pairs in their output forms with other, segmentally identical words, specified for H(L) (the L of the contour is not realized in these monosyllables), e.g., *gó* [H] ‘*go*’ vs. *gò* [L] ‘FUT’, and *bín* [H] ‘to have been in a location’ vs. *bìn* [L] ‘PST’. Note the prosodic parallelism between the minimal pair *gò* vs. *gó* in Table 1 (Pichi).

Guyanese Creole also has disyllabic tonal minimal pairs. Two examples follow (stressed syllable in bold, HL lexical tone indicated by a rising-falling circumflex): (1) ***pa****kît* /**Ø**-HL/ ‘packet’ → [H-H] vs. (2) ***pâ****kit* **/HL**-Ø/ ‘pocket’ → [HL-H] and (1) ***flo****wâ /***Ø**-HL/ ‘flour’ → [H-H] vs. (2) ***flô****wa /***HL**-Ø/ ‘flower’ → [HL-H]. For want of space, I shall not delve into the complex rules formulated by Devonish (2002, 86–95) as well as Devonish and Thompson (2010, 10–11) to account for the divergent realization of these word-tone patterns in phonological brackets to the right of the arrow. What is relevant is that the output tone patterns after the arrow show a pitch contrast between forms (1) and (2). Note that both sets of minimal pairs feature stress on the penultimate syllable, showing that the assignment of tone is independent of the assignment of stress.

#### 2.2.2. Tonal Processes and Grammatical Tone

Guyanese Creole does not seem to have a similarly broad use of grammatical tone as Pichi. Compounding and reduplication nevertheless show parallels with Pichi (Devonish and Thompson, 2010, 13–57). Like in Pichi, the formation of compounds (1) and reduplications (2) (Table 4) involves tonal derivation: The lexical HL tone is deleted in the non-final component (the dependent), while the final component (the head) retains its lexical HL tone (Devonish and Thompson, 2010, 11). Note that in long vowels the contour is spread out across both moras hence *láàng* ‘long’.

**TABLE 4:** Tonal derivation of compounds and reduplications in Guyanese Creole.

	Component 1	Component 2	Compound/Reduplication
(1)	*shâp* /HL/ ‘shop’	*frônt* /HL/ ‘front’	*shap-frônt* /Ø-HL/ ‘shop front’
	*blâk* /HL/ ‘black’	*pê.pa* /HL/ ‘pepper’	*blak-pê.pa* /Ø-HL.Ø/ ‘black pepper’
(2)	*láàng /*HL/ ‘long’	*láàng /*HL/ ‘long’	*laang*-*láàng* /Ø-HL/ ‘long here and there’

The prosodic pattern of Guyanese Creole (and Pichi) compounds and reduplications is therefore the opposite of that found in British English, where the first component receives stress and the second is deaccentuated, i.e., ***shop****-front*. I have provided evidence elsewhere that the prosodic features of compounding found in Guyanese Creole, Pichi, and other Afro-Caribbean English-lexifier Creoles conform to an areal pattern found across West Africa (Yakpo, 2012).

#### 2.2.3. Residual Tone

Guyanese Creole also features ‘residual tone’. The term goes back to Berry (1972) and has been employed by some to characterize the occurrence of lexical, phrasal, or grammatical tone in specific semantic fields, and grammatical and pragmatic functions in Afro-European creoles otherwise characterized by stress systems (e.g., Todd, 1980; Granda, 1986; Smith and Adamson, 2006). In Guyanese Creole, residual tone is found in the formation of ideophones. The features of residual tone differ from those of the tone system described in section 2.2.1–2.2.2 in the following way: (1) There are two distinct tone heights, which differ from the HL contour described above, suggesting an /H, L/ or /H, Ø/ contrast; (2) There is no evidence for a quantity contrast (i.e., stress) in the ideophones covered here.

Ideophones depict sensory imagery pertaining to sensations like motion, visual appearance, texture, and feelings (Dingemanse, 2018). They also tend to be structurally marked cross-linguistically, for example through the presence of phonemes that are rare in other word classes of the same language, or the presence of lexical tone in a prosodic system otherwise characterized by stress, as in Guyanese Creole (All examples in this section are from Hubert Devonish, pers. comm. Tones in ideophones are marked). Example (8) contains the ideophone *pím.pím* /H.H/ ‘remained quiet, did not respond verbally when a response might have been expected’, with two successive H tones. Example (9) features the ideophone *kìtàkàtà* /L-L-L-L/ ‘hectically’, which bears L tones throughout. The ideophone *brámbrámbrám /*H-H-H/ ‘with a rumbling noise’ (10) features three successive H tones. All three ideophones consist of (lexicalized) duplications or triplications, a common feature in African creoles and non-creoles (see section 2.1.1).


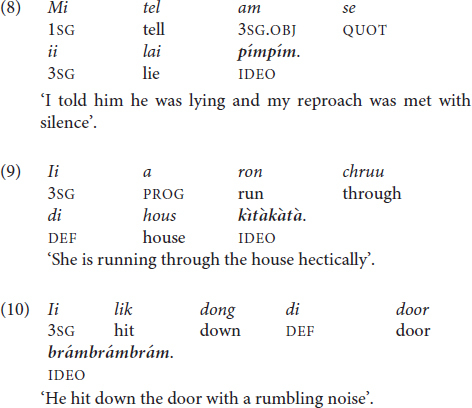


Residual tone possibly also occurs in some degree-modifying adverbs, where an extra-high tone expresses focal emphasis together with the adverb. The multifunctional word *sotil* is a clause introducer with the meaning ‘until’ in time clauses like (11). When the clause introducer occurs at the end of a clause in an ‘unfinished utterance’, as in (12), it expresses emphasis and meanings like ‘a lot’ or ‘excessively’. In the latter instance, *sótíl* always bears extra-high pitch on both syllables. The African English-lexifier creoles feature both uses of the corresponding form *(só)té(é)* as well, including the use of extra-high tone and final-vowel lengthening (see section 4.2) (for an example sentence in Pichi, see Yakpo, 2019a, 277). The phenomenon has also been described for Sranan and African creoles under the term ‘register raising’ (Smith and Adamson, 2006; see section 3.3 and section 3.4). Only an acoustic analysis can eventually clarify whether the extra-high tone in a context like (12) instantiates lexical tone or a purely intonational use of pitch.


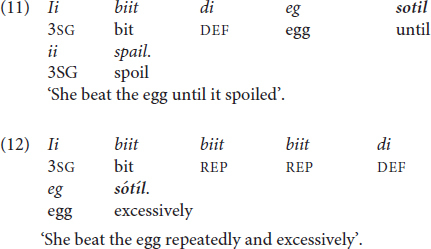


## 3. Afro-European Contact Prosodic Systems Show an Areal Distribution Across the Atlantic

The analyses in section 2 have shown the existence of tone and stress systems in the same linguistic family, as well as mixing between the two prosodic types. I will now argue that Afro-European creoles and colonial varieties show an areal distribution across the Atlantic basin (section 3.4), which is roughly coterminous with the presence of tone in the east (section 3.1) and stress in the west (section 3.3). Transitional systems combining features of tone and stress are found in the areal buffer zone of the Caribbean (section 3.2). When the social and linguistic composition of an ecology changes, contact languages and varieties can shift from tone to stress systems and vice versa (section 3.5).

### 3.1. African Creoles and Colonial Varieties, and American Maroon Creoles Have Tone Systems

Tone systems typify all creoles and European colonial varieties (the varieties of English, French, Spanish, and Portuguese) spoken in the tonal ecologies of Africa for which detailed phonological data is available. Tone is one of the most conspicuous typological features across Africa (Maddieson, 2005). Tone was therefore naturally imposed on the prosodic systems of creoles and colonial varieties spoken in Africa. Tone systems are also found in isolated Maroon creoles of the Americas, which probably retained tone systems from earlier times (Rivera Castillo and Faraclas, 2006 provide a first typological comparison of African and Maroon creole systems with African non-creole systems).

All African English-lexifier creoles have been described as tonal. Krio (e.g., Berry, 1970; Hancock, 1971; Nylander, 1984; Finney, 2004), Pichi (Yakpo, 2019a), and Nigerian Pidgin (Faraclas, 1996) have been analyzed in detail, showing the presence of equipollent 2-tone systems with fully specified H and L tones, fixed word-tone patterns and tonal minimal pairs. Most English-derived words have a culminative and obligatory H. Polysyllabic lexemes with more than one H or no H at all are fewer and are mostly found in words with an African etymology. All African English-lexifier creoles make use of grammatical tone. We find identical or similar instantiations of grammatical tone like tone deletion and replacement during compounding and reduplication in Pichi (section 2.1.2), Nigerian Pidgin (Faraclas, 1996, 251–252), Krio (Finney, 1993), Cameroon Pidgin (Nkengasong, 2016, 36–37), and Ghanaian Pidgin (Huber, 2003). Case functions in personal pronouns are expressed by tonal contrasts (e.g., Nigerian Pidgin, Faraclas, 1991). For the better studied languages Nigerian Pidgin and Pichi, word-level and phrase-level processes have been described including downstep, tone-spreading, deletion, polarization (the OCP-triggered assignment of an opposite, polar tone, to an adjacent morpheme/syllable), and pitch or register raising. Preliminary analyses of my field data suggest that most of the lexical, grammatical, and phrasal functions of tone identified for the other African English-lexifier creoles are also found in Ghanaian Pidgin and Cameroon Pidgin.

Tone systems are also found in the insular Gulf of Guinea Portuguese-lexifier Creoles Forro (Maurer, 2008) and Angolar (Maurer, 1995), spoken in São Tomé, Lung’le (Agostinho and Hyman, 2021), spoken in Príncipe, and Fa d’Ambô, spoken in Annobón. Fa d’Ambô and Lung’Ie have been analyzed as languages with a privative /H, Ø/ contrast, based on the stress contrast of Portuguese. The value Ø is generally realized as [L]. In Fa d’Ambô, for example, we find stress-to-tone mapping between lexifier and creole forms such as the following (H-toned and stressed syllable in bold here and thereafter): *fa****la* /**Ø-H/ ‘say’, from Port. *fa****lar*** ‘say’, and ***mo****sa* /H-Ø/ ‘woman’, from Port. ***mo****ça* ‘girl’ (Zamora, 2010).

The analysis of Lung’Ie, in turn, shows a privative /H, Ø/ contrast between the three word-tone patterns /H/, /Ø-H/, and /H-Ø/. The prosodic lexicon is etymologically stratified. Portuguese-sourced nouns have a culminative H tone, e.g., ***pá****ta* /H-Ø/ ‘duck’ from Port. ***pa****ta*. African-sourced words are, by contrast, toneless, and bear L output tones, e.g., *ugbododo /*Ø-Ø-Ø-Ø/ → [L-L-L-L] ‘precipice’. Agostinho and Hyman (2021, 88) explain the somewhat unexpected feature of the latter stratum by the resolution of the prosodic clash between the minority African lexicon (with non-culminative, non-obligatory H) and the Portuguese-sourced majority lexicon (with culminative H tone due to stress-to-tone mapping). Moreover, Lung’Ie has not been in much contact with tonal African adstrates for several centuries because of Príncipe’s geographical isolation as an island. Idiosyncratic outcomes and innovations are to be expected during prosodic mixing due to differing social histories, and should not be seen as unique to creoles (see also Good, 2009). There is no evidence for stress in Lung’Ie (Agostinho and Hyman, 2021, 81–86) and due to the absence of similarly detailed acoustic analyses, it is difficult to substantiate claims that Forro and Fa d’Ambô employ stress in addition to tone (e.g., Traill and Ferraz, 1981; Zamora, 2010).

By contrast, the family of Upper Guinea Portuguese-lexifier creoles of Cape Verde (Kabuverdianu) (Swolkien, 2015), Guinea-Bissau (Kriyol) (Chapouto, 2014), and Senegal (also called Kriyol by its speakers) (Biagui, 2012) have all been analyzed as languages with stress, not tone. Lang (2009) and Jacobs (2010) provide convincing lexical and structural evidence for a founder role of non-tonal Wolof (Atlantic) at a crucial period in the development of Upper Guinea Creole in the 15th century. Other Atlantic languages that were probably represented in the creole founder population (e.g., Fula, Seereer, and Joola) and are still spoken alongside the creole in Guinea Bissau and Senegal also have stress-only systems. This aligns the Upper Guinea creoles prosodically with other non-tonal languages spoken in adjoining parts of West Africa. Further, non-tonal Portuguese has been spoken alongside these creoles by the descendants of founder populations for several centuries (Jacobs, 2010, 302–307), hence much longer than other European languages in Africa. Nevertheless, there is equally strong linguistic evidence for an input of the tone languages Manding (Mande) and Temne (Atlantic) into Proto-Upper Guinea Creole (Quint and Tavares, 2019). Alternatively, the Upper Guinea Creoles could therefore also have completed a shift from tone to stress due to the prolonged absence or marginalization of African tone languages in the ecology, just like most creoles of the Caribbean. The presence of residual tone in Cape Verdean has indeed been suggested by Macedo (1979, 132–134), though not corroborated by acoustic analyses.

Crucial support for the areal distribution of prosodic systems across the Afro-American Atlantic comes from the presence of tone systems in African varieties of English, French, Spanish, and Portuguese, i.e., of the very lexifiers of the creoles. West African varieties of European colonial languages like Nigerian English (Gussenhoven and Udofot, 2010) and Ghanaian English (Criper-Friedman, 1990) have been analyzed as privative systems with a two-way /H, Ø/ contrast and fixed word-tone patterns. In both varieties, English-sourced content words feature a culminative and obligatory H. The syllable with primary stress in the British English cognate receives an H, as in ***mem****ber /*H-Ø/ → [H-L]. Monosyllabic function words that are unstressed in British English are toneless and L-toned in the output, e.g., *of* /Ø/ → [L], *a* /Ø/ → [L], and *he* /Ø/ → [L]. Nigerian and Ghanaian English both also feature rightward H tone spreading in utterance-medial positions (e.g., ***mem****ber* /H-Ø/ → [H-H]), as well as downdrift.

Central African French and Equatorial Guinean Spanish have been analyzed as equipollent /H, L/ systems with fully specified tone and no stress (Bordal Steien and Yakpo, 2020). In Central African French, an /H/ is realized on the final syllable of every content word, thus replicating the most frequent position of phrasal stress at the word level in European French. Other syllables receive an /L/. Central African French has two fixed word-tone patterns, namely /L/ and /(L)H/, e.g., *ce* /H/ ‘this’, *le* /L/, *sentir* /L-H/ ‘feel’). Equatorial Guinean Spanish, in turn, has four word-tone patterns, namely /L(-L)/, /(L-)H/, /(L-)H-L/, and /(L-)H-L-L/, e.g., *desde* /L-L/ ‘since’, *yó* /H/ ‘1SG.SBJ’, *porqué* /L-H/ ‘why?’, *clase* /H-L/ ‘class’, *película* /L-H-L-L/ ‘film’.

Central African French and Equatorial Guinean Spanish both have tonally distinguished minimal pairs in the category of function words, e.g., Equatorial Guinean Spanish *tú* /H/ ‘2SG.SBJ’, *tu* /L/ ‘2SG.POSS’. The tone of Central African French personal pronouns is not only lexically specified but also unpredictable on the basis of French stress, e.g., *ils* /H/ ‘3PL.SBJ.M’ vs. *il* /L/ ‘3SG.SBJ.M’. Equatorial Guinean Spanish also has H tone spreading and downdrift, e.g., *jóvenes* /H-L-L/ ‘youths’ → [H-H-H]. Tone systems apparently also characterize other African Romance varieties, among them the French varieties of Côte d’Ivoire (Boutin and Turscan, 2009) and Mali (Bordal and Skattum, 2014). The data on African varieties of Portuguese is not conclusive (e.g., Santos, 2019 for Angolan Portuguese). But it would be unusual if these varieties had stress-only systems, since most of them are spoken in tonal ecologies.

Besides the creoles and colonial varieties spoken in Africa, the Maroon creoles of the Americas for which we have conclusive data feature tone systems. Maroon creole languages are spoken by the descendants of Africans who liberated themselves from European enslavement and established independent polities in areas that remained geographically secluded until the 20th century. Maroon creoles therefore also remained relatively isolated from (non-tonal) European superstrates and creoles until quite recently (e.g., non-tonal Dutch and Sranan in Suriname). Saramaccan (traditionally spoken in the Amazonian interior of Suriname) has a two-height contrast, fixed word-tone patterns, and tonal minimal pairs (Good, 2009). Most Portuguese- and English-sourced words feature a privative /H, Ø/ contrast. /H/ is borne by the syllable that bears stress in the lexifier; compare ***wó****mì* /H-Ø/ ‘man’ (<Port. ***ho****mem* ‘man’) and *à****kí* /**Ø-H/ ‘here’ (<Port. *a****qui*** ‘here’). African-sourced words are fully specified for tone and feature an equipollent /H, L/ specification. In addition, a phonologized extra-H tone /!H/ is found in mostly African-sourced ideophones (Good, 2006, 20), thus constituting a third tone height (see 4.3). African words also have more diverse patterns, e.g., *lɛ̀gɛ̀dɛ̀* /L-L-L/ ‘lie (noun)’ and *tótómbòtí* /H-H-L-H/ ‘woodpecker’. There are also numerous tonal processes in Saramaccan, including H tone spreading and raising, and plateauing (Rountree, 1972; Good, 2004). Like in the African English-lexifier creoles, personal pronouns are inflected by tonal ablaut to express case functions, e.g., *mì* ‘1SG.SBJ/POSS’ vs. *mí* ‘1SG.OBJ/EMP’ (McWhorter and Good, 2012, 42).

The related Surinamese Maroon creole Ndyuka also has a two-way height contrast including tonal minimal pairs, e.g., *tàkì* /L-L/ ‘quotative complementizer’ vs. *tákì* /H-L/ ‘say’ (Huttar and Huttar, 1994, 5). Compounds are created via the same tonal derivation as in the African English-lexifier creoles, i.e., *káw* /H/ ‘chew’ and *bón* /H/ ‘bone’ → *kàw-bón* /L-H/ ‘chewed-bone(s)’ (Huttar and Huttar, 1994, 373). The analysis by Hualde and Schwegler (2008) of the prosodic system of the Spanish-lexifier Maroon creole Palenquero spoken in the town of Palenque de San Basilio (Colombia) also indicates the presence of a tone system, when it is stated that ‘accented’ syllables *consistently* carry high (contour) pitch’ (Hualde and Schwegler, 2008).

Instead of seeing the tone systems described in this section as exceptional they should be understood as typical instantiations of source language agentivity in ecologies dominated by tone languages. Such tone systems develop through the three mechanisms of stress-to-tone mapping, paradigmatization, and idiosyncratization (see section 4).

### 3.2. Some American Varieties Combine Stress With Tone

A second group of contact languages and varieties combines stress with tone in various ways. The resulting mixed systems include (sparse) tone systems of the type encountered in Guyanese Creole and Papiamentu (see below for the latter), in which tone and stress co-occur throughout the lexicon. Further, they extend to systems with fully specified ‘residual tone’ (Berry, 1972) in specific semantic fields and in specialized functions in prosodic systems otherwise characterized by stress alone, or both stress and sparse tone (e.g., Guyanese Creole, section 2.2).

Sranan (Suriname), for example, makes use of stress alone in the majority of its lexicon with characteristic effects like lengthening of stressed syllables, shortening of unstressed ones and consonant gemination, e.g., *pa****pa*** ‘father’ → [p**pa**], *wo****wo****yo* ‘market’ → [w**wo**yo] (van der Hilst, 1988, 51–54). Sranan ideophones, however, have fixed H or L tones, e.g., *píí* /H-H/ ‘quietly’, *pétépété* /H-H-H-H/ ‘thoroughly’ vs. *tjùbùm* /L-L/ ‘with a plopping sound’ (Smith and Adamson, 2006).

The use of lexical tone in addition to stress has also been posited for Tobagonian in the distinction between the grammatical and pragmatic functions of personal pronouns (James, 2003), e.g., *dèm* /L/ ‘3PL’ vs. *dém* /H/ ‘3PL.EMP’ (for parallels with Krio/Pichi, see section 4.2). In the absence of acoustic evidence that such uses of pitch in Tobagonian are indeed tonal, and not concomitants of stress and intonation, this is, however, difficult to verify.

Haitian Creole differs from its lexifier European French in that individual words all bear lexical stress on the final syllable (Cadely, 1994; for acoustic evidence, see Kalkhoff, 2018). In Haitian, the prosodic constituent is therefore not the accentual phrase as in European French, but the prosodic word, which is also the domain of attribution of lexical tone. In addition, the Haitian post-nominal determiner is stressed and consistently high-pitched, e.g., *mayi-****a*** ‘maize-DET’. Kalkhoff (2018) proposes that this is in emulation of the H tone of the corresponding post-nominal determiner in Gbe, Haitian’s main substrate cluster (Kalkhoff, 2018), i.e., *bl*ǐ*-****á*** ‘maize-DET’ (own knowledge). Further, in some basilectal varieties of Haitian (e.g., rural varieties that incorporate fewer features from the French superstrate than urban varieties), word stress is apparently replaced by word-final high pitch alone, thus mirroring the tone systems of African varieties of French (Brousseau, 2003, 132; see section 3.1). Sylvain (1936; cited in Gooden, 2003, 193) mentions the existence of tonal minimal pairs in distinguishing intensive from attenuative meanings in reduplications, e.g*., píké-píké /*H-H-H-H/ ‘very pricking’ vs. *pìkè-pìkè* /L-L-L-L/ ‘slightly pricking’. There are thus indications that Haitian has residual tone, and has merged aspects of the stress-only system of its lexifier European French with the tone systems of its African substrates, prompting Kalkhoff (2018) to call it ‘mixed’.

Besides Guyanese Creole (section 2.2), the Iberian-lexifier creole Papiamentu (Netherlands Antilles) is prosodically fully mixed in the sense that the acoustic properties of tone and stress co-occur and are generalized across the entire lexicon (Devonish, 1989, 60; Rivera-Castillo, 1998; Rivera-Castillo and Pickering, 2004; Remijsen and Van Heuven, 2005). Papiamentu has a tone system that combines lexical word stress with lexical tone and numerous tonal processes, including downstep and polarization. The vowels of stressed syllables are longer and louder than unstressed ones, and unstressed syllables tend to be more centralized (i.e., more schwa-like) than stressed ones. Some analyses postulate an /H, L/ equipollent system with full lexical specification of tones (Rivera-Castillo, 1998; Kouwenberg, 2004). According to Remijsen and Van Heuven (2005), Papiamentu has a privative /HL, Ø/ contrast. Syllables specified for Ø are realized as lower than the H of the HL lexical tone or they carry an LH intonational pitch accent that signals focus. Further, the lexical HL tone can, but need not coincide with the stressed syllable. The position of the lexical HL is also unpredictable in a large number of words. In Joubert (1991), there are over two hundred tone-stress minimal pairs, see ex. (1–3) below. Further, tone distinguishes (1) disyllabic verbs from (2) disyllabic nominals and is therefore also used for derivation (see Kouwenberg, 2004, 66–69 for more instances of grammatical tone). Examples follow (stressed syllable in bold, HL lexical tone indicated by a rising-falling circumflex): (1) ***lo****râ* /**Ø**-HL/ ‘to turn’ (verb), (2) *lo****râ*** /Ø-**HL**/ ‘turned’ (participle), (3) ***lô****ra* /**HL**-Ø/ ‘parrot’.

Few Afro-European creoles have been studied as extensively with regard to prosody as Papiamentu. It is therefore possible that systems with stress and (sparse) tone as well as residual tone are far more common in the Americas than meets the eye. Some of the stress-only systems described in section 3.3 and others not mentioned here could therefore turn out to be mixed as well.

### 3.3. Other American Varieties Combine Stress With African Intonational Features

Many Afro-Caribbean creoles and American varieties of European colonial languages spoken by African-descended majorities feature stress-only systems without lexical and morphological tone. But ‘suspicious’ features raise the possibility of a tonal past and an areal switch from tonal or mixed to stress-only systems (see section 3.5 and 5.2 for further discussion). Relevant prosodic features of some of these creoles and colonial varieties are discussed in the following.

Gooden (2003) produces evidence that the English-lexifier Creole Jamaican makes use of lexical stress, not lexical tone. But the nature of pitch movements associated with word-level stress and intonational pitch accents is unlike that of its lexifier British English (e.g., an H^∗^L on stressed English syllables vs. an H+L^∗^ on stressed syllables in Jamaican). Equally, compounding involves morphological stress placement on the rightmost morpheme like in Guyanese Creole (see section 2.2.2), which is reminiscent of compounding in tonal Pichi (see section 2.1.2), but unlike English, where the first morpheme is stressed. Further, the prosodic rhythm of Jamaican (Thomas and Carter, 2006) and Guyanese Creole (Devonish, 2002, 96–97) is syllable-timed. Syllable timing means that the duration of each syllable is more or less equal, unless there is some form of pragmatic marking. Syllable timing gives Jamaican an auditory impression that prompted earlier (English-speaking) observers to mistakenly classify Jamaican as tonal (e.g., Lawton, 1963). The prosodic rhythm of the lexifier British English is, by contrast, stress-timed. Phonetic effects to achieve optimal prosodic rhythm in British English are the lengthening of vowels in stressed syllables, the reduction of vowels in unstressed syllables, e.g., *police* [plis], as well as vowel laxing, e.g., *sane* [sein] vs. *sanity* [saniti] (Ciszewski, 1999, 30). Syllable timing, rather than stress-timing generally appears to be a hallmark of African tone languages (Gut et al., 2001) and is therefore very likely to be a tonal carry-over (see Bloomquist et al., 2015, for a summary of similar arguments with respect to Jamaican, Jamaican English, and African American English).

Many Caribbean creoles, whether they have been analyzed as tonal or not, also show pitch-related intonational features found across tonal Africa. These include utterance-level declination, which parallels downdrift and is widely attested in tonal Africa (Yip, 2002, 262–263), as well as ‘register raising’ (assignment of extra-high pitch to the entire relevant constituent, not just the stressed syllable) for focal emphasis (for Guyanese Creole, Trinidadian English Creole, and Bajan, see Sutcliffe, 2003; for Sranan, see Smith and Adamson, 2006; for Pichi, see Yakpo, 2019a, 55–57). A further, seemingly pan-Caribbean intonational feature with parallels in the tonal substrates is an utterance-final fall in wh-questions (Sutcliffe, 2003), which corresponds to ‘lax question intonation’, a Macro-Sudan areal feature of Africa (Güldemann, 2018, 481). Lax intonation has also been described for the Gulf of Guinea Portuguese-lexifier Creoles (Agostinho et al., 2019).

Colonial varieties of English, French, Spanish, and Portuguese spoken by African-descended majorities in the Americas also show intonational features that differ in often substantial ways from the colonial varieties spoken by European-descended populations. Caribbean varieties of English have a reputation for their ‘melodic intonation’, an auditory impression that is, again, occasioned by their greater degree of syllable-timing, un-English pitch contours associated with stress, and a greater range of pitch variation across stressed and unstressed syllables alike (see Wells, 1982).

Speakers of European French also think that Caribbean French has an ‘accent chantant’ because speakers of the latter place stress on individual words, not accentual phrases, again like Haitian. They also tend to stress the first syllable of multisyllabic words in addition to the last, and may stress clitics and prepositions, something that speakers of European French do not normally do (Pustka, 2007).

In the same vein, authors have commented upon, though often not described in detail, the peculiar prosodic characteristics of rural varieties of Spanish and Portuguese spoken in countries with large African-descended populations and by isolated communities of African origin. Popular (vernacular) Brazilian Portuguese, for example, has more utterance-internal pitch accents than European Portuguese with a frequent alternation between and H^∗^ and L^∗^, and ‘tonal events not linked to stressed syllables’ (Frota and Vigário, 2000, 11).

Rao and Sessarego (2016) provide a detailed acoustic and phonological analysis of aspects of the prosody of Afro-Bolivian Spanish. They mention, among other features not found in other Bolivian Spanishes, an obligatory and fixed LH pitch contour or H level pitch over stressed syllables. In European and European-influenced American varieties word-level pitch contours can, by contrast, be significantly altered by intonation (see Hualde and Prieto, 2015 for an overview). Butera et al. (2020) arrive at a similar conclusion with respect to the Afro-Peruvian variety of Spanish spoken in the province of Chincha, Peru. There is cursory evidence for the existence of similar prosodic features in other American Spanish varieties as well, which require further substantiation (e.g., Choco Spanish and Congo, see Lipski, 2007).

The analyses of numerous American ‘stress-only’ varieties remain somewhat inconclusive. It is well possible that many feature residual tone or constitute mixed tone-stress systems as well. Either way, many of their distinct prosodic characteristics are very likely to result from the incorporation of pitch features of the tonal substrate languages once spoken by the African creators of these varieties.

### 3.4. The East to West, Africa to the Americas, Tone to Stress Areal Continuum of Prosodic Systems

Table 5 presents the areal east-west, Africa-Americas, tone-stress continuum of prosodic systems in the languages surveyed in section 3.1–3.3. Their classification is based on information contained in the sources cited there. The prosodic features *tone*, *residual tone*, *stress* in the headers of the three central columns are checked against the column captioned *languages*. The symbols + and – indicate the presence or absence of features. When in parentheses (+), evidence for the feature is anecdotal in the literature, i.e., not corroborated by acoustic evidence and detailed phonological analysis. The rightmost column provides details of the three checked features, with numbers (1)–(4) referring to the following characteristics discussed in section 2.1–2.2 and section 3.1–3.3: (1) type of prosodic system and tonal inventory, (2) phrase-level and utterance-level tonal or pitch-related processes, (3) aspects of grammatical tone, (4) aspects of intonation and prosodic rhythm. The *languages* column contains linguistic groupings and individual varieties. These are, in turn, grouped in the *Group* column in the following way:

**TABLE 5:** The areal continuum of Afro-European contact prosodic systems.

Group		Languages	Tone	Residual tone	Stress	Description of prosodic features
**East** **(tone)**	1	Tonal substrate and adstrate languages of Africa	+	–	–	(1) Mainly 2T and 2T3; equipollent (e.g., /H, L/) and privative (e.g., /H, Ø/) (2) Lots of word-level and phrasal tonal processes, incl. everything in Group 2 (3) Lots of grammatical tone, incl. everything in Group 2 (4) Lax question intonation (Macro-Sudan languages) and rising intonation (e.g., some Bantu languages); syllable timing
	2	African English-lexifier creoles (Krio, Pichi, Nigerian Pidgin, Cameroon Pidgin, Ghanaian Pidgin)	+	−	−	(1) 2T and 2T3 systems; equipollent /H, L/, privative /H, Ø/; /!H/ in pragmatically salient functions (degree words, ideophones)
		Gulf of Guinea Portuguese-lexifier creoles of Africa (Forro, Angolar, Lung’Ie, Fa d’Ambô)	+	−	−	(2) Downstep; H-tone raising, spreading, deletion, floating; OCP/polarization; pitch raising for emphasis
		Colonial varieties of European languages spoken in Africa (Nigerian English, Ghanaian English, Central African French, Equatorial Guinean Spanish)	+	−	−	(3) Compounding and reduplication; tonal inflection of personal pronouns; tone-conditioned allomorphy (Pichi); portmanteau morphemes with contour tones (African English-lexifier creoles)
		Maroon creoles of the Americas (Saramaccan, Ndyuka, Palenquero)	+	−	−	(4) Lax question intonation next to rising question intonation; syllable timing
	3	Papiamentu, Guyanese Creole, and possibly other Caribbean creoles	+	+	+	(1) 2T *and* stress; privative /H, Ø/; words with residual tone possibly have equipollent /H, L/; /!H/ in pragmatically salient functions (degree words, ideophones); contrastive stress (2) Downstep; H-tone raising, spreading, deletion, polarization (Papiamentu); pitch raising for emphasis (3) Compounding and reduplication; derivation (Papiamentu) (4) Lax question intonation next to rising question intonation; syllable timing
	4	Caribbean English-lexifier creoles (Sranan, Tobagonian, and possibly others), Caribbean French-lexifier creoles (Haitian, and possibly others)	–	+	+	(1) Contrastive word stress; no tone in the majority lexicon; residual tone with privative /H, Ø/ or equipollent /H, L/ in specific functions and fields (e.g. ideophones and reduplication) (2) Utterance-level and/or phrase-level pitch downtrends but no tonal downstep; pitch raising for emphasis (3) Possible grammatical (residual) tone in compounding and reduplication (4) Lax question intonation next to rising question intonation; syllable timing
	5	Caribbean English-lexifier creoles (Jamaican, Trinidad Creole English, Bajan, and others)	−	(+)	+	(1) Contrastive word stress; no tone in the majority lexicon; no firm evidence for residual tone but likely in some
		Upper Guinea Portuguese-lexifier Creoles (Kriyol, Kabuverdianu)	−	(+)	+	(2) Utterance-level and/or phrase-level pitch downtrend but no tonal downstep; pitch raising for emphasis
		Colonial varieties of European languages spoken by African-descended populations and many vernacular colonial varieties of the Americas (Caribbean French and English, Popular Brazilian Portuguese, Afro-Bolivian and Afro-Peruvian Spanish, Choco Spanish, and possibly others)	−	(+)	+	(3) Morphological stress (e.g., stress shift in compounds and reduplications); no grammatical tone (4) Lax question intonation next to rising question intonation; syllable timing
**West (stress)**	6	Stress-only lexifier and superstrate varieties of Europe and colonial (standard) varieties of European-descended populations of the Americas (e.g., European French and English, Argentinian Spanish, and others)	–	–	+	(1) Contrastive word-level and phrase level stress; no (residual) tone (2) Utterance-level and/or phrase-level pitch downtrend; no tonal downstep (3) Morphological stress; no grammatical tone (4) Rising question intonation; stress-timing (English, Dutch) and syllable-timing (Spanish, French)

The eastern pole (Group 1) at the top of Table 5 is represented by the tonal substrates and adstrates of Africa. The western pole (Group 6) at the bottom is represented by the stress-only superstrates and lexifiers (English, French, Spanish, Portuguese, and Dutch) spoken in Europe and by largely European-descended populations in the Americas. Group 2–5 prosodic systems emerged from contact between Group 1 (tone) and Group 6 (stress) systems.

Table 5 shows a tone-stress cline from Group 1 to 6 languages with a gradual decrease in tonal features and a concomitant increase in stress-related features. Group 2 creoles and colonial varieties are exclusively tonal (section 3.1). Group 3 features the mixed systems of Papiamentu and Guyanese Creole (section 2.2 and 3.2) that combine stress and privative tone in all of their lexicon, additionally feature residual tone and many but not all of the tonal features of Group 2 languages detailed in the rightmost column. Group 4 languages (section 3.2) feature stress-only systems in most of their lexicon but there is evidence for residual tone in ideophones and some grammatical functions (e.g., compounding and reduplication). Group 4 languages share some pitch-related phrasal and intonational features reminiscent of Group 1 and 2 tone languages (downtrend, pitch or register raising, lax question intonation).

Group 5 languages have stress-only systems (section 3.3). The evidence for residual tone is anecdotal, hence (+) in the corresponding column. However, the evidence is more conclusive that Group 5 languages have incorporated African intonational features in which they overlap with Group 1–4 languages (e.g., lax question intonation in some languages, pitch or register raising, and syllable timing).

Groups 2–5 are idealized types and we should expect many more variations, gradations, and idiosyncrasies than captured by Table 5. It is also possible that future research reveals that many languages now in Group 4 and 5 have more tonal features than presently known.

### 3.5. Areal Switches Are Common in Creole Prosodic Systems

In section 3.1–3.4, I argued for the existence of an areal continuum of contact prosodic systems from Africa to the Americas. Two diachronic scenarios are thinkable on the basis of the demographic (African-descended majorities) and linguistic (tone-dominant ecologies) evidence in relation to the areal continuum summarized in section 3.4. One scenario would suggest that Group 4 and 5 languages in Table 5 have always featured stress-only systems but incorporated substratal tonal features in their prosodic systems. However, I tend to think that Group 4 and 5 languages that evolved in ecologies with overwhelming African-descended majorities, once had full-blown tone systems like those of Group 2 (see section 3.1). Areal convergence with European stress-only lexifiers and superstrates and other stress-only languages in the ecology (e.g., the Indic languages of the Caribbean, see below) would have then led to the replacement of tone by stress systems and the retention of sparse and residual tone in some varieties.

In languages spoken in ecologies with somewhat less of a demographic dominance of African populations vis-à-vis European populations, as well as the right social factors (e.g., a slightly more porous social stratification of Africans and Europeans), stress and tone could have co-evolved right from the start (e.g., Papiamentu as well as Spanish and Portuguese colonial varieties of the Americas).

A switch from tone to stress has explicitly been claimed by Barth (2016) for Sranan (Suriname). Barth argues on the basis of historical phonology that Sranan once had a tone system like its closest relatives, the Maroon creoles Ndyuka and Saramaccan, and then lost tone through contact with Dutch. The survival of African-style systems of lexical and grammatical tone in the Maroon creoles makes it plausible that many other American creoles and colonial varieties started out as tonal and later shifted to stress-only prosodic systems (see Alleyne, 1980; Devonish, 1989). With the end of the European slave trade in the 19th century, the proportion of L1 speakers of African tone languages began to decline. Throughout the 20th century, socio-economic change led to the partial erosion of racialized social stratification, while formal education in the standardized European varieties of colonial languages was expanded (Yakpo, 2015; also Hackert, 2019, 225).

In the wake of such social transformations, the American ecologies came to be dominated by patterns of societal multilingualism involving creoles and the stress-only superstrates English, Spanish and Dutch. In several cases (Guyana, Suriname, Trinidad), non-tonal Indic adstrates also played an important role in the ecology. The influence of Bhojpuri, for instance, has contributed to changes in the pitch associated with stressed syllables in Trinidad Creole English (Gooden et al., 2009, 419–420). It is possible that source-language agentivity in Bhojpuri and other non-tonal languages of Trinidad besides English (e.g., Portuguese, see Ferreira, 2006) also contributed to the demise of tone in Trinidad Creole English.

The opposite areal switch from stress to tone is, by contrast, a possibility in the trajectory of Krio, the English-lexifier creole of Sierra Leone. In one account, Krio is seen as an offshoot of Western Maroon creole of Jamaica brought to Sierra Leone in the late 18th century by African-descended returnees (Smith, 2017). Jamaican is a stress-only language today (see section 3.3) and contemporary Krio is a tone language without stress (see section 3.1). If Western Maroon creole had already acquired a stress system in Jamaica by the time it arrived in Sierra Leone, then Proto-Krio would have jettisoned stress for tone due to adstratal influence from speakers of African tone languages. Most prominent among these adstrates were Yoruba, Gbe, Mende, Temne, and Manding (Hancock, 1971; for the historical background, see Huber, 1999, 59–74).

The commonness of switches between types of prosodic systems is corroborated by evidence from regions other than the Afro-Atlantic. The Tibeto-Burman family is presently split half-way between languages that employ tone and others that use stress. Tonal Tibeto-Burman languages are found in a prosodic linguistic area encompassing tonal Tai-Kadai, Hmong-Mien and Chinese languages (Ratliff, 2015).

The reverse switch involving ‘tonoexodus’ (Matisoff, 1973) is also attested. If Proto-Afro-Asiatic was tonal, as suggested by some (see Wolff, 2018), then the tonal Cushitic and Omotic subbranches of Afro-Asiatic retained tone during millennia of contact with tonal Nilo-Saharan. The Proto-Semitic subbranch of Afro-Asiatic therefore probably lost tone along the way. The Cushitic languages Kemant and Khamtanga therefore appear to have lost tone through contact with (Ethiopian) Semitic (Appleyard, 1991, 10). In northern Norway (Jahr, 1984; Bull, 1995) and southern Finland (Bruce, 2004), the superstrates Norwegian and Swedish, which employ both stress and tone (Kristoffersen, 2000), underwent substratal and adstratal transfer from the stress-only languages Sami and Finnish. The resulting contact varieties of Norwegian and Swedish have lost tone and feature stress-only systems.

There is therefore ample evidence that tonogenesis and tonoexodus are cyclical and complementary processes rather than one-way streets (see also Matisoff, 1973). There is no reason to exclude creoles from these cross-linguistic tendencies (see section 5.2 for further discussion).

## 4. Contact Prosodic Systems With Tone Emerge Through Three Cognitive-Typological Mechanisms

In the preceding sections, I have argued that the distribution of tone and stress is areal across the Afro-American Atlantic. Tone predominates in the east (Africa), stress in the west (Americas), and transitional systems cluster in the areal buffer zone of the Caribbean. Given that the tonal creoles and colonial varieties all have lexifiers with stress-only systems, it is useful to take a closer look at the emergence of tone systems during the encounter of non-tonal lexifiers and tonal substrates and adstrates.

Ratliff (2015, 258) cautions against *broad* explanations for tonogenesis in language contact because they fail to explain ‘exactly how tones were either transferred to—or stimulated to develop in—previously atonal languages under contact’. Bordal Steien and Yakpo (2020) propose three *specific* cognitive-typological mechanisms in the genesis of contact prosodic systems with tone, namely: (1) stress-to-tone mapping (section 4.1), (2) paradigmatization (section 4.2), and (3) idiosyncratization (section 4.3).

### 4.1. Stress-to-Tone Mapping

Through the mechanism of stress-to-tone mapping speakers of tone languages create a tone system in the contact language or variety by building on perceptual analogies between the phonetic realizations of stress and tone (Bordal Steien and Yakpo, 2020, 23–26).

For one, high or rising pitch is a consistent correlate of stress besides duration, loudness, and vowel quality in the European lexifiers, e.g., in French (Jun and Fougeron, 2002), British English (Morton and Jassem, 1965), and Spanish (Hualde and Prieto, 2015). Speakers of tone languages are perceptually also more sensitive to pitch variations than to other acoustic cues of stress. Speakers of Mandarin Chinese selectively perceive the higher pitch of stressed syllables in English, rather than vowel length, loudness, and vowel quality. This makes pitch the primary cue for distinguishing stressed from unstressed syllables for native Chinese speakers (Wang, 2008). In other words, the pitch contour of a stressed syllable is reinterpreted as a tonal contour by tone language speakers and other cues of stress are ignored.

As a result, the position of H in the creoles coincides with primary stress placement in the cognate forms of the lexifier, e.g., Pichi, *go* → *gó* /H/, ***car****penter* → ***kyá****pìntà* /**H**-L-L/, ***en****ter* → ***ɛ́n**tà* /**H**-L/, *for****get*** → *fɔ̀**gɛ́t*** /L-**H**/, *under****stand*** → *ɔ̀ndà**stán*** /L-L-**H**/. H tone in Pichi is therefore culminative and obligatory in English-sourced content words, just like primary stress placement is in English. Low tones are found on all syllables that do not bear stress in the corresponding English source word. Stress-to-tone mapping is attested in all the Afro-European creoles and colonial varieties covered in section 3.1 and other contact languages not mentioned so far, e.g., in the African Arabic-lexifier creoles Juba Arabic (Nakao, 2013) and Kinubi (Gussenhoven, 2006). Stress-to-tone mapping also characterizes the prosodic systems of Euro-Asian creoles and colonial varieties that arose from the encounter of stress-only superstrates and tonal substrates and adstrates (for an overview of several varieties, see Ng, 2011 and Lim, 2012; for a detailed study of Hong Kong English, see Wee, 2016).

### 4.2. Paradigmatization

The second mechanism in the creation of contact prosodic systems is paradigmatization (Bordal Steien and Yakpo, 2020, 26–28). Paradigmatization occurs by default when stress patterns of the non-tonal lexifier are mapped onto tone patterns in the contact languages and varieties. In the case of lexicon sourced from African tone languages, tone classes can be carried over into the contact language without prior stress-to-tone mapping, as in the case of Mende and Yoruba items in Krio described further below. But there may be contact-induced adaptation even with African-sourced tonal words, see e.g., 3.1, on Lung’Ie. The resulting tone classes therefore largely mirror corresponding stress and tone classes in the European and African input languages, respectively (see section 2.1.1).

Besides the replication of prosodic structures from source languages, paradigmatization may also regularize functional paradigms. The pronominal system of all African English-lexifier creoles is divided into two series. One series expresses subject and possessive case and invariably bears an L tone, e.g., *yù* ‘2SG.SBJ/POSS’ (<*you*), *wì* ‘1PL.SBJ/POSS’ (<*we*) and *dɛ̀m* ‘3PL.SBJ/POSS’ (<*them*). The other series assumes the syntactic and pragmatic functions of object case and emphasis and is exclusively H-toned, e.g., *yú* ‘2SG.OBJ/EMP’, *wí* ‘1PL.OBJ/EMP’, and *dɛ́m* ‘3PL.OBJ/EMP’ (e.g., *you*, *we*, and *them*). Hence, it is *à sí* ***yú*** *yɛ́stàdé!* ‘I saw you yesterday’, but not ^∗^*à sí* ***yù*** *yɛ́stàdé!* The English-sourced pronouns have therefore undergone paradigmatization in the creoles in order to fit into the L-toned and H-toned case paradigms, respectively. Paradigmatization in the tonal English-lexifier creoles of Africa shows numerous overlaps with that of the tonal English-lexifier creoles of Suriname (e.g. Saramaccan, see section 3.1), suggesting it was already present in the proto-creole(s).

Contact varieties whose lexifiers have fewer stress patterns show correspondingly fewer word-tone patterns. Phrase-final stress in European French has been converted into word-final stress in the tonal African varieties of French (Bordal Steien, 2015; Bordal Steien and Yakpo, 2020). Paradigmatization has only rendered two word-tone patterns in Central African French on the basis of the corresponding European French potential for stress placement (see section 5.2 for further discussion).

### 4.3. Idiosyncratization

The third mechanism, idiosyncratization, leads to the emergence of arbitrary word-tone patterns, paradigms and constructions. While paradigmatization creates tonally regular forms and paradigms, idiosyncratization therefore creates tonally irregular ones including idiosyncratic grammatical and syntactic tone rules. Idiosyncratization may occur through any combination of the factors of substratal and adstratal imposition, the operation of cross-linguistic tendencies (e.g., interactions of tone with consonant or syllable type), and language-specific constructionalization and grammaticalization, whether through contact or not.

An example follows of idiosyncratization in the word-tone patterns of content words. The African English-lexifier creoles have a considerable stock of English-sourced words in which H tone does not coincide with English primary stress. Examples from Krio/Pichi are ***wa****ter* → *wà****tá*** /L-**H**/, ***trou****sers* → *trɔ̀**sís*** /L-**H**/, ***pro****perty* → *prɔ̀pà****tí*** /L-L-**H**/, ***hos****pital* → *ɔ̀s**pí**tùl* /L-**H**-L/. Such words have undergone tone shift and no longer exhibit a prosodic parallelism with their English etymons (for the background to these changes, see Devonish, 2001). Tone shift therefore constitutes idiosyncratization vis-à-vis the English input.

A further example of idiosyncratization in the lexicon follows from Krio and Pichi. Both creoles have a set of degree-modifying and quantifying words, see Table 6. (1, 2) are English-sourced lexicalized reduplications (<*little, big*); (3) is of unknown origin [but see the back formation *sótíl* < *^∗^(so) till* in Guyanese Creole, ex. (11, 12)]; (4) is probably Igbo-sourced (< s*ọ̀*s*ọ̀* ‘only’). The exclamation mark before the /!H/ tone signals that it is extra-high, hence a notch above the usual H tone register. H tone raising is conventionalized with these words, as is the lengthening of the final vowel in *sótéé* (3).

**TABLE 6:** Tonal idiosyncratization in lexical words (Krio and Pichi).

	Item	Tone pattern	Example
(1)	*lílí(lí)* ‘little, tiny’	/!H!H/	*dì wàtá tú* ***lílílí*** ‘the water (is) too little’
(2)	*bíbí* ‘big, huge’	/!H!H/	*wán* ***bíbí*** *hós* ‘a huge house’
(3)	*sótéé* ‘excessively’	/!H!H!H/	*à rɔ́n* ***sótéé*** ‘I ran excessively’
(4)	*sósó* ‘only’	/!H!H/	***sósó*** *mɔ́nìn tɛ́n* ‘really early in the morning’

Idiosyncratization has therefore rendered a set of tonally arbitrary forms with respect to stress-to-tone mapping, and these are grouped in a specific semantic field. The use of /!H/ and lengthening for degree modification are probably both iconic processes (see Thompson, 2018, for a possibly universal prevalence of H tone in the sound symbolic lexicon) (also see section 5). But I have shown that the similar process of pitch or register raising for emphasis also occurs in other African and Caribbean English-lexifier creoles, see 2.1.2–2.2.3 and 3.1–3.3.

The presence of tone-conditioned suppletive allomorphy in Pichi is an example of the idiosyncratization of grammatical tone (see section 2.1.2), even if the conditioning feature of an obligatory tonal contour over adjacent tone-bearing units draws on an areally widespread model (Yakpo, 2019b, 217–218).

Contact tone systems can only incorporate the material provided by their input languages during stress-to-tone mapping. In combination with the two other mechanisms of paradigmatization and idiosyncratization, prosodic contact nevertheless leads to autonomous outcomes, particularly in the creoles in contrast to the European colonial varieties (see section 5.2).

## 5. Creole Prosodic Systems Are Areal, Not Simple

I now revisit arguments for claims that tone is eliminated or simplified in creoles (see section 1). A comparison with tonal African non-creoles allows the conclusion that the tone systems of Afro-European creoles are neither particularly simple nor typologically divergent in other ways (section 5.1). Instead, creole prosody undergoes regular typological change and areal convergence (section 5.2).

### 5.1. Creole Tone Systems Are No Simpler Than African Non-creole Tone Systems

The hypothesis that creolization involves prosodic simplification has at least two subjacent assumptions: (i) Creolization is seen to involve the elimination of features that are difficult for adult learners (for a thematic overview, see Siegel, 2004), and tone is viewed as ‘particularly hard to master during untutored second language acquisition’ (Sessarego, 2020, 4).

This assumption is logically flawed, for it does not distinguish between the two psycholinguistic dominance relations of recipient language agentivity and source language agentivity (see Bordal Steien and Yakpo, 2020), and the two corresponding transfer types of borrowing and imposition (van Coetsem, 1988, 2000). Phonology (van Coetsem, 2000), and prosody in particular (Matras, 2009, 231–33), are the most stable domains of a natively acquired grammar. So if any domain gets transferred to a contact language from its input languages at all, *it will be prosody*, and in ecologies dominated by tone speakers, *it will be tone* (Bordal Steien and Yakpo, 2020). The assumption of tone loss is also Eurocentric because it takes for granted that stress systems, which happen to characterize all European lexifiers and superstrates, constitute the fallback during prosodic contact.

(ii) Creoles can have tone systems, but these systems are assumed to be simpler than non-creole tone systems (see the sources cited in the opening paragraph of section 1). Assumption (ii) deserves some attention. In the face of irrefutable evidence for the existence of creole tone systems, it is less categorical than the tone loss hypothesis, yet aligns with the ‘creoles-are-simpler-than-other-languages’ hypothesis (for an overview and a critique, see Ansaldo and Matthews, 2007).

In the following, I address the characteristics of tone in Afro-European contact prosodic systems with respect to: (1) tonal inventories (i.e., the number of distinctive tones), (2) the existence of tonal minimal pairs, (3) the number of word-tone patterns, (4) and the nature of tonal processes and rules, comparing these with features of tonal non-creole languages of Africa.

Tone systems with two heights, whether equipollent /H, L/ or privative /H, Ø/, as in the Afro-European creoles and colonial varieties are the most common ones across a huge swath of West Africa and all of West Central Africa as far south as Angola (Wedekind, 1985; Hyman et al., 2020). These were the principal home regions to the millions of Africans enslaved and deported to the Americas by the Europeans (Eltis, 2001). Their languages therefore constituted the most important substrates to the creoles and colonial varieties that developed in the Americas and in the West African littoral region. A two-way contrast is also cross-linguistically the most common type beyond Africa (Maddieson, 2013) and tonogenesis almost always produces a binary contrast between two tone heights (Hyman et al., 2020).

Further, a significant number of relevant African substrate and adstrate languages feature restricted three-height systems termed 2T3 height systems by Hyman (2018, 208) (2 input vs. 3 output heights, e.g., /H, L, Ø/). In such systems, the presence of an additional (e.g., mid or extra-high) tone is, for example, conditioned through a constructional tone rule or the segmental structure of its tone-bearing unit and therefore predictable and not lexical in *sensu stricto* (as in Yoruba, see Akinlabi, 1985).

The conventionalization of extra-high tone in Krio and Pichi degree-modifying words is an example of such a restricted use of a third height in the creoles (see section 4.3). In contrast, systems with three and more *lexical* tones have marked regional distributions. In West Africa, these are principally found in the Kru languages and the adjacent contact zones, the Mabia (Gur) languages, and the Nigerian-Cameroonian plateau region. None of these areas were pre-dominant home regions of enslaved Africans as far as the historical records are concerned (see Eltis, 2001).

A second argument encountered for classifying creole and other contact prosodic systems as simpler is the supposedly low number of tonal minimal pairs. This is claimed by McWhorter (1998), an assertion contradicted in later work by the same author’s listing of over twenty tonal minimal pairs in Saramaccan, many of which are multisyllabic (McWhorter and Good, 2012, 39). Matisoff (2001, 304) argues that largely monosyllabic languages are particularly ‘tone (and phonation) prone’ for marking semantic and grammatical distinctions, due to the sparsity and interdependence of segmental material in the word.

Conversely, the functional load of tone in the lexicon will be reduced in languages with longer words. It therefore comes as no surprise that tone languages in which two or more syllables predominate should be characterized by fewer tonal minimal pairs in the lexicon than logically possible. This is the case in Akan (Kwa, Ghana), for example, (e.g., *pàpá* ‘father’, *pápá* ‘good’, *pàpà* ‘fan’, ^∗^*pápà*; own knowledge), the Narrow Bantu languages (Hyman et al., 2020), Cushitic (Mous, 2009), Chadic (Rolle, 2018), and, last but not least, the Afro-European creoles and colonial varieties.

Thirdly, African tone languages of all linguistic lineages feature restrictions on the distribution of tones and the number of word-tone patterns. Susu (Mande, Guinea) only has the three tone patterns H, H-L, and L-H over mono- and disyllabic nouns (nouns with more syllables are not very common) (Green et al., 2013). Two tone patterns, H and L-H, cover ninety per cent of the lexicon of Bambara (Mande, Mali) (Dumestre, 2003, 22).

Further, it is very common for African substrates and adstrates of Afro-European creoles to have lexical strata displaying specific prosodic behaviors, often as a result of the imposition of native prosody on loan lexicon, just like in the creoles. In the two-height system of Mende (Mande, Sierra Leone), loanwords bear a cumulative H on the penultimate syllable, irrespective of the original position of the H-toned or stressed syllable in the source language. Native vocabulary is not subject to such a restriction (Leben, 1977; cited in Clements and Ford, 1979:201). In the three-height tone system of Ewe (Kwa, Ghana, and Togo), European loanwords have a cumulative H on the stress-bearing syllable in the source language, other syllables are L-toned, e.g., *à****bó****lò* ‘bread’ (<Port. ***bo****lo* ‘cake’, incl. nominal prefix *à-*), *à**k**ɔ́ntà* /L-**H**-L/ ‘arithmetic’ (<Port. ***co****nta* ‘account’), *sù****kú****ù* /L-**H**-L/ ‘school’ (<Eng. *school*, incl. epenthetic vowel /ù/), ***dú****kù* /**H**-L/ ‘scarf’ (<Dutch *doek* ‘scarf’, incl. paragogic vowel /ù/). The tonal integration of European loanwords in Akan (Apenteng and Amfo, 2014) and Gã (Kropp Dakubu, 1999), two other languages of the Ghanaian littoral zone, proceeds along similar lines.

Kikongo (Narrow Bantu, Congo, and DRC) has a privative /H, Ø/ system and H tone is obligatory and cumulative in much of the lexicon. French loans bear a word-final cumulative H due to stress-to-tone mapping, just as in Central African French, e.g., *kà****dó*** /Ø-**H**/ ‘present’ (<Fr. *cad****eau***), *prɛ̀z**ìdɔ̃́*** /Ø-Ø-**H**/ ‘president’ (<Fr. *prési****dent***). By contrast, word-final H is rare in the African-sourced lexicon, compare *ndúùmbù* /H-Ø-Ø/ ‘spices’, *èntsàngálà* /Ø-Ø-H-Ø/ ‘basket’ (Donnelly, 1982).

Besides loanwords, ideophones also display special phonological (and morphosyntactic) characteristics cross-linguistically (Dingemanse, 2018). In African non-creoles and creoles alike, ideophones exhibit additional tone heights (e.g., extra-high) and tone types (e.g., contour tones instead of level tones alone), as well as idiosyncratic tone patterns. In the 2-tone language Temne (Mel, Sierra Leone), ideophones generally take an H or LH (composite) tone where other word classes take an L or HL tone. This renders tonal minimal pairs like *gbâŋ* /HL/ ‘manufacture a tool’ vs. *gbǎŋ* /LH/ ‘extremely old (IDEO)’ (Kanu, 2008).

In the two-tone system of Kisi (Atlantic, spoken in Guinea and Sierra Leone), contour tones are not restricted to final syllables in ideophones and are also found on initial syllables, unlike in other word classes, e.g., *kpîngmgbí* /HL-H/ ‘darkly’ (IDEO) (Childs, 1988). There are also many more H tones and extra-H tones than L tones in Kisi ideophones, which is the opposite of the distribution in non-ideophonic word classes, e.g., *kpáng* /!H/ ‘tightly, carefully (IDEO)’.

Beyond the ideophonic lexicon, restrictions on the number, types and position of tones are common throughout tonal Africa (for an overview, see Downing, 2010). This is again no different from the creoles. A good many Bantu tone systems are characterized as sparse because they feature a privative /H, Ø/ contrast. The single /H/ toneme is obligatory and culminative, and its position predictable (Odden, 1988; Gussenhoven, 2004, 34–35; Hyman, 2011b, 235). But restrictions are also found in systems with full specification of lexical tones. In Akan, vowel height and the place of articulation of consonants determine the distribution of H and L tones in disyllabic verbs, e.g., *pìrá* /L-H/ ‘hurt’ (first vowel is high and followed by a sonorant) vs. *kásà* /H-L/ ‘speak’ (first vowel is non-high) (Welmers, 1973, 118).

A fourth argument for simplification is the claim that there is no grammatical tone in creoles (McWhorter, 2005, 13–14). ‘Grammatical tone’ is poorly delimited in the first place, making it difficult to distinguish from tone sandhi phenomena, intonation, phrasal tonology, even lexical tone (Rolle, 2018, 3), and the terminology is unclear (Rolle, 2020, 71). If we assume the definition of grammatical tone as a tonological operation restricted to the context of a specific morpheme or construction (Rolle, 2018), there is a vast range of phenomena that can be subsumed under the label in Africa (e.g., polar tone in Yoruba, see Akinlabi and Liberman, 2000; and constructional tone in Kalabari, see Harry and Hyman, 2014). Some African tone languages have much grammatical tone, others less (Hyman et al., 2020). In comparison, tonal Afro-European creoles neither feature abundant nor particularly sparse grammatical tone. Uses discussed in this study include the expression of pronominal case functions by tonal minimal pairs in all tonal English-lexifier creoles, e.g., in Pichi (section 2.1.2) and in Saramaccan (McWhorter and Good, 2012, 42).

Tonal inflection in the pronominal paradigm in addition to segmental inflection for number and case is common in West African tone languages, including Ewe (Duthie, 1996, 53), Akan (Dolphyne, 1996, 109–110), and Edo (Uchihara, 2010). I have shown that creole grammatical tone also extends to tonal derivation in compounding and reduplication, constructional tone and OCP, as well as tone fusion and contour tone formation (see section 2.1.2). The creoles also have phrasal tone rules of tone spreading, downdrift, dissimilation, assimilation, and tone insertion, all of which are shared with African tone languages (Hyman and Schuh, 1974; Yip, 2002). These rules have been investigated in detail for creoles like Pichi (see section 2.1 and the references there), Saramaccan (Good, 2004, 2006, 2009), and Lung’Ie (Agostinho and Hyman, 2021, 77–80). There is good reason to assume that similar rules exist in other tonal Afro-European creoles of Africa and the Americas.

In sum, the prosodic systems of Afro-European creoles do not differ from the systems of African non-creole languages in any consistent and sufficient way to qualify them as simpler in terms of tonal inventories, the existence of tonal minimal pairs, the number of tone patterns, and the nature of tonal processes and rules. Instead, creole tone systems fit snugly into the areal patterns attested in countless variations throughout tonal Africa.

### 5.2. Creole Prosodic Systems Undergo Regular Typological Change and Areal Convergence

Contact prosodic systems acquire their properties from a typological matching exercise between features of the input languages in a specific linguistic ecology (Mufwene, 1996, 2001; Aboh and Ansaldo, 2007). The acoustic and phonological realizations of tone imposed by the adstrates and substrates can initially only be grafted on the prosodic patterns of the lexical material provided by lexifier stress patterns.

The more typologically compatible the input systems are, the more tonal features will be utilizable. In other words, if a creole evolved from contact between the Mande language Dan (Côte d’Ivoire, Liberia) with 5 tone heights and the neighboring Kru language Krahn with 3 tone heights, tone-to-tone mapping should allow a higher number of tonal features to be utilized. Claims that tone is reduced in function or lost in African contact languages that have emerged from contact between *tone languages* should be taken with a grain of salt, due to the absence of detailed analyses (e.g., Heine, 1978, 221 and the sources cited there). The only comprehensive study of the prosody of the Kikongo cluster (Congo, RDC, Angola), for example, concludes that the Bantu-based contact language Kikongo-Kituba ‘has merely accelerated certain tendencies inherent in the Kikongo pitch system’ (Donnelly, 1982, 343).

The prosodic systems that emerge under the typological constraints of stress-to-tone mapping will therefore not look like the tone system of a language like Guébie (Kru, Côte d’Ivoire), with its above-average number of tonal features utilized by West African standards. Guébie has five contrasting tone levels and abundant grammatical tone (Sande, 2018). Clements and Rialland (2007, 70–74) argue that in all areas of West and East Africa with a similarly high functional load of tone, lexical and grammatical tone was not borrowed from neighboring languages. Instead there was an areal diffusion of monosyllabicity, the structural prerequisite for such a functional proliferation of tone. None of the tonal creoles of Africa have a predominantly monosyllabic template, so it is not surprising that they have 2T (two heights) or 2T3 (2 input, 3 output heights), not five-tone systems. However, there is no reason to assume that diachronic change of a creole through areal pressure toward monosyllabicity should not produce additional tone heights and more active grammatical tone than is already the case.

Afro-European creoles are ‘late arrivals’ in their respective linguistic ecologies in the sense that they owe part of their lineage to exogenous Indo-European lexifiers. The prosodic systems of Afro-European creoles therefore continue to align themselves over time with other languages in their respective ecologies according to regular areal dynamics (for an overview of these dynamics, see Raymond, 2017, 3–5). Since convergence takes time, typological inconsistencies may persist in the prosodic systems of creoles vis-à-vis those of their tonal (Africa) or stress-only (Americas) areal cohabiters.

For example, a large part of the lexicon of Ghanaian Pidgin is sourced from English and therefore features a cumulative H due to stress-to-tone mapping. By contrast, the Ghanaian Pidgin adstrates Akan, Gã, and Ewe have a much smaller European-sourced lexicon with a cumulative H (see section 5.1). Such an inconsistency between Ghanaian Pidgin and the non-creoles in the Ghanaian ecology is not caused by creole distinctiveness, but by differences in the size of the European-derived prosodic lexicon. Likewise, the persistence of (residual) tone in Caribbean creoles and areally unusual prosodic layering in an African creole like Lung’Ie (African-sourced words are toneless) means that these languages have not (yet) fully aligned themselves with their adstrates and/or superstrates. These differences are gradual, and can progressively narrow down due to continuing areal convergence, or stabilize as innovations that bring additional typological diversity to an ecology.

Such a role of time depth in the areal diffusion of prosodic features is fundamentally different from the idea that creoles are young and have not yet had time to accumulate (tonal and morphological) complexity in their grammars (e.g., Bickerton, 1988, 274–278; Lightfoot, 2006, 7; McWhorter, 2007, 4–5). The latter view is a trope rooted in 19th century linguistic evolutionism (for an epistemological deconstruction, see Krämer, 2013; McElvenny, 2021). The results of this study instead suggest that creoles and colonial varieties undergo regular cycles of shift from one part of the typological spectrum (e.g., tone) to another (e.g., stress) and vice versa, without an *a priori* assumption of simplification or complexification.

In the scenarios covered here, such shifts are contact-driven and result in prosodic convergence between unrelated and typologically dissimilar languages cohabiting the same ecology, and prosodic divergence between related languages inhabiting different ecologies. Prosodic convergence and divergence are reflected particularly well in the range of prosodic systems found in the family of Afro-Caribbean English-lexifier Creoles, with its large geographical spread across diverse linguistic ecologies in Africa and the Americas.

## 6. The Idea of Creole Simplicity Is a Chimera

The prosodic systems of Afro-European creoles and colonial varieties form an areal continuum across the Afro-Atlantic from Africa to the Americas, roughly corresponding to tone in the east and stress in the west. Transitional systems are found in the areal buffer zone of the Caribbean, where tone and stress-only systems have converged in various ways. Numerous pitch-related phenomena found in American creole languages and colonial varieties of the Americas that exclusively feature stress today suggest the existence of tone or mixed tone-stress systems before the shift to stress-only systems.

I have identified and described three mechanisms involved in the emergence of contact prosodic systems with potential for generalization to prosodic contact scenarios beyond the Afro-Atlantic. These are stress-to-tone mapping, paradigmatization, and idiosyncratization. The label ‘simple’ neither captures these mechanisms themselves, nor the tone systems they engender.

The argument that creoles are simpler than non-creoles is based on the notion of ‘bit complexity’ (DeGraff, 2001, 284–285), which boils down to ‘more overt material = more complex’. Bit complexity has been criticized as a simplistic and arbitrary criterion of little heuristic value for measuring linguistic complexity (Aboh and DeGraff, 2017, 14–20; Newmeyer, 2021).

Nevertheless, even from the perspective of bit complexity, *creole tone systems are more complex* than those of the colonial varieties of English, French, Spanish, and Portuguese spoken in Africa (section 3.1 and section 4). This is due to social factors that impede the same amount of innovation and areal diffusion of tonal features to the colonial varieties as to the creoles. The colonial varieties are heavily standardized, are usually acquired in classrooms, and are predominantly used in formal settings. The proportion of speakers who regularly use the colonial varieties is small and limited to social classes with access to secondary and tertiary education (for French in Africa, see e.g., Mufwene, 2011). The natural evolution of European colonial varieties, including that of their prosodic systems, is therefore severely constrained (Yakpo, 2020, 133–134). By contrast, the creoles have been evolving without state-sanctioned standardization and are primarily spoken by urban working class and rural populations, many of whom have little formal education and limited exposure to the colonial varieties. The creoles could therefore acquire many more autonomous prosodic features through areal diffusion from substrate and adstrate languages than the colonial varieties.

The idea of creole simplicity is a chimera. Research should rather focus on the roles played by genealogy, areal typology, cognition, and social factors in shaping the fascinating diversity of specific language contact outcomes, as I have attempted here with respect to prosodic systems.

## Data Availability Statement

The raw data supporting the conclusions of this article will be made available by the authors, without undue reservation.

## Ethics Statement

The studies involving human participants were reviewed and approved by the Human Research Ethics Committee, The University of Hong Kong. Written informed consent for participation was not required for this study in accordance with the national legislation and the institutional requirements.

## Author Contributions

KY designed the work, collected and analyzed the primary data, assembled secondary data from other sources, conducted the qualitative analyses, and wrote the manuscript.

## Conflict of Interest

The author declares that the research was conducted in the absence of any commercial or financial relationships that could be construed as a potential conflict of interest.

## Publisher’s Note

All claims expressed in this article are solely those of the authors and do not necessarily represent those of their affiliated organizations, or those of the publisher, the editors and the reviewers. Any product that may be evaluated in this article, or claim that may be made by its manufacturer, is not guaranteed or endorsed by the publisher.

## Glossary

**Table:**

=	Clitic morpheme boundary
1	First person
2	Second person
3	Third person
2T	Two tone system
2T3	2 input vs. 3 output tone heights
H	High tone
L	Low tone
M	Mid tone
HL	High-low contour tone
LH	Low-high contour tone
H-L	Separates tone-bearing units (syllables), e.g., *kátà* /H-L/ ‘scatter’
!H	Extra-high tone
↓H	Downdrifted or downstepped high tone
/H/	Input (phonological) tone
[H]	Output (phonetic) tone
/HL/ → [H]	Input /HL/ becomes output [H]
H*	High intonatonal pitch accent
L*	Low intonational pitch accent
ó	High level tone
ò	Low level tone
ô	High-low (falling) contour tone
ǒ	Low-high (rising) contour tone
C	Consonant
COMP	Complementizer
DEF	Definite article
EMP	Emphatic
Eng.	English
F	Feminine gender
FUT	Future tense
Fr.	French
IDEO	Ideophone
INDF	Indefinite article
IPFV	Imperfective aspect
M	Masculine gender (in glosses)
NEG	Negative
OBJ	Object case
OCP	Obligatory Contour Principle
PL	Plural number
Port.	Portuguese
POSS	Possessive case
POT	Potential mood
PROG	Progressive aspect
PRF	Perfect tense-aspect
PST	Past tense
QUOT	Quotative complementizer
REP	Repetition
SBJ	Subject case
SBJV	Subjunctive mood
SG	Singular number
V	Vowel
V́	H-toned vowel
V̀	L-toned vowel
